# Wedge resection versus segmentectomy in peripheral clinical stage IA lung cancer concerning ground-glass opacity

**DOI:** 10.1007/s00595-025-03137-4

**Published:** 2025-09-22

**Authors:** Atsushi Hata, Yutaro Sato, Takamasa Ito, Takayoshi Yamamoto, Yusuke Otani, Yuichi Sakairi, Takekazu Iwata

**Affiliations:** Division of Thoracic Surgery, Chiba Cancer Center, 666-2, Nitona-cho, Chu-o-ku, Chiba, 260-8717 Japan

**Keywords:** Sublobar resection, Wedge resection, Segmentectomy, Lung cancer, Clinical stage IA

## Abstract

**Purpose:**

Recent randomized controlled trials have shown the non-inferiority of sublobar-to-lobar resection for small peripheral non-small cell lung cancer (NSCLC); however, whether wedge resection (WR) or anatomical segmentectomy (SG) is superior remains unclear. We hypothesized that ground-glass opacity (GGO) is associated with the outcomes of WR and SG.

**Methods:**

Between 2010 and 2022, 219 consecutive patients with clinical stage IA peripheral NSCLC who underwent sublobar resection for frailty at our institution were retrospectively reviewed. Based on the high-resolution computed tomography findings, the tumors were classified into two groups: part-solid (GGO (+)) and solid (GGO (−)). The long-term outcomes were compared between the WR and SG groups.

**Results:**

In the part-solid group (*n* = 124; median CTR, 0.62), WR was equivalent to SG in terms of 5-year disease-free survival [DFS] (98% vs. 91%; *p* = 0.2) and recurrence rate (0% vs. 4.3%;* p* = 0.3). In the solid tumor group (*n* = 95), WR was inferior to SG in terms of the 5-year DFS (43% vs. 80%; *p* < 0.01) and recurrence rate (32% vs. 3.7%; *p* < 0.01).

**Conclusions:**

In our study population, WR was not inferior to SG for part-solid tumors. However, for solid tumors, the long-term outcomes of SG are superior to those of WR.

**Supplementary Information:**

The online version contains supplementary material available at 10.1007/s00595-025-03137-4.

## Introduction

Sublobar resection is widely recognized as an intentional therapeutic option, especially for peripheral non-small cell lung cancer (NSCLC), after the CALGB140503 and JCOG0802 trials. These trials showed that sublobar resection was not inferior to lobar resection for small-sized (≤ 2 cm) peripheral NSCLCs with respect to survival [1, 2]. However, whether wedge resection (WR) or anatomical segmentectomy (SG) is superior remains unclear. The report of the CALGB study regarded these procedures as the same sublobar resection and did not mention the reason for their choice; it then compared lobectomy with sublobar resection, including both SG and WR. On the other hand, the JCOG regarded only SG as a sublobar resection in their non-inferiority studies for lobectomy. Therefore, we examined whether WR is equivalent to SG, and if not, which procedure offers better long-term survival outcomes, particularly among frail patients. WR and SG in this study are thought to be passive indications based on the fact that lobectomy has recently been the standard therapy. The consolidation tumor ratio (CTR) has been widely used to predict tumor aggressiveness [3, 4] in recent studies, and it has been shown that the presence of a ground-glass opacity (GGO) component, even if slight, leads to better outcomes relative to cases without GGO [3, 5]. We hypothesized that WR, which is less invasive than SG, would be more suitable for GGO tumors. To clarify the feasibility of both types of sublobar resection, we compared WR and SG in clinical stage IA (c-stage IA) peripheral NSCLCs with or without GGO in frail patients.

## Methods

**Fig. 1:**
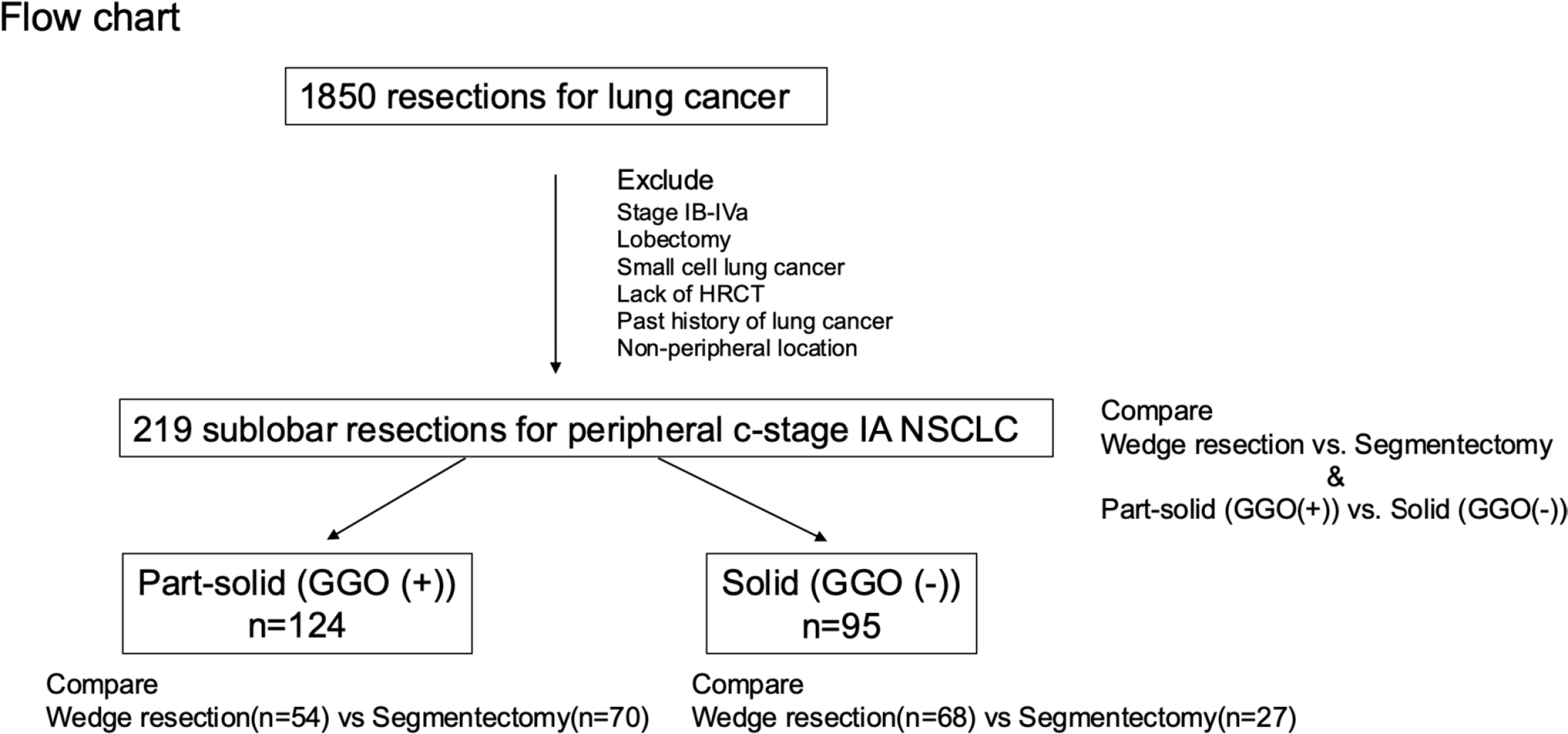
Flowchart of the study. Two hundred nineteen sublobar resections were assigned in this study. We compared wedge resection vs. segmentectomy and part-solid (GGO (+)) vs. solid (GGO (−)). Then, wedge resection vs. segmentectomy was compared in the part-solid and solid subtypes

This study was approved by the Institutional Review Board of Chiba Cancer Center (approval codes: R06-109 and R05-104), and the requirement for informed consent was waived because of the retrospective nature of this study. Clinical data were collected from the institutional cancer registry database and outpatient follow-up visits. The records included preoperative patient characteristics, disease status (including CTR), operative procedures (WR or SG), pathological data, and follow-up data. Among 1850 consecutive lung cancer resections between 2010 and 2022 at Chiba Cancer Center, 219 patients with c-stage IA peripheral primary NSCLCs who underwent sublobar resection were included in this study. The 8th edition of TNM staging in Lung Cancer of the International Association for the Study of Lung Cancer was adopted in this study. Clinical staging was determined using high-resolution computed tomography (HRCT), fluorine-18 fluorodeoxyglucose positron emission tomography CT (PET-CT), and contrast-enhanced magnetic resonance imaging of the brain. Patients were excluded if they had a history of lung cancer, non-peripheral tumor, tumor in situ, malignant pleural effusion, or dissemination revealed intraoperatively or if they lacked HRCT. Peripheral tumors were defined as those located within the outer third of the lung field on HRCT images. The following items were included in the analysis: age, sex, pulmonary function, presence of interstitial lung disease (ILD), history of cardiovascular disease (CVD), tumor size, CTR, c-stage, surgical procedure (WR or SG), pathological type, pleural invasion (pl), lymphovascular invasion (ly and v), outcomes (postoperative complications, 30-day mortality, recurrence [locoregional or distant], disease-free survival [DFS], lung cancer-specific survival [LCSS], and overall survival [OS]). ILD was defined as abnormalities compatible with bilateral fibrosis on CT, such as peripheral reticular opacities, or pathological findings of interstitial changes, such as fibrotic foci. CVD includes aortic aneurysms, ischemic heart disease, hypertrophic cardiomyopathy, and arterial thrombosis. Several thoracic surgeons and pulmonologists have reviewed the CT scans. Patients were divided into part-solid (tumor with GGO) and solid (tumor without GGO) groups based on HRCT. Clinical characteristics and survival were compared between the two groups, and the prognostic factors were analyzed. Postoperative complications were evaluated using the Clavien–Dindo classification. Locoregional recurrence was defined as tumor relapse in the ipsilateral thorax, which included the resection margin of the lung or bronchus, hilar lymph nodes, mediastinal lymph nodes, and malignant pleural effusion, according to the previous JCOG 0802 trial [1]. A flowchart of the present study is shown in Fig. 1.

### Indications and techniques for sublobar resection

In this study, sublobar resections were mainly performed for frail patients who could not tolerate or in whom lobectomy would be considered high risk because all resections in this study were performed before the CALGB140503 and JCOG0802 studies revealed the efficacy of intentional sublobar resection. The decision regarding the type of sublobar resection (i.e., WR or SG) is usually a combination of the patient’s performance status and tumor characteristics (CTR, location, etc.). WR is generally preferred over SG for patients with an increased risk based on comorbidities. For SG, we begin with hilar dissection to clear tissues and lymph nodes (LNs) from segmental vessels and bronchi, which are subsequently knotted or stapled. The segmental borders were then divided using staplers. Lung inflation was performed after stapling the segmental bronchus if the segmental borders were not easily identifiable. Systemic lymph node dissection was often omitted because many patients were intolerable in this study. For WR, the tumor was palpated and grasped, and staplers with adequate safety margins were used to obtain a 2-cm parenchymal margin around the tumor or a margin equivalent to the tumor diameter. Most patients treated with WR and SG undergo video-assisted thoracic surgery (VATS) in a minimally invasive manner.

### Statistical analysis

Data were analyzed using JMP Pro (ver. 16, SAS Institute Inc., Cary, NC, USA). The Mann–Whitney *U* test was used to compare continuous variables between the two groups, while Fisher’s exact test was used for categorical variables. Survival curves were estimated using the Kaplan–Meier method, and differences in survival times between the two groups were calculated using a log-rank test. Propensity score matching was performed to reduce patient selection bias in the part-solid and solid groups. The propensity score was the probability of segmentectomy estimated using a logistic regression model that included age ≥ 80 years, sex, smoking status, FEV1.0% < 70%, presence of ILD, history of CVD, and c-stage for DFS. These variables were selected from the univariate analysis of WR and SG characteristics. After propensity score matching, DFS was compared between the two groups for each subtype (part-solid and solid). A Cox hazard model was used to identify the independent prognostic factors. All reported *p* values were 2-sided, and *p* values < 0.05 were considered to indicate a statistically significant difference. Independent prognostic factors for shorter survival were analyzed using a Cox proportional hazards regression model after adjusting for confounding variables.

## Results

### Comparison of preoperative patient characteristics between surgical procedures

Of the 219 patients, 122 underwent WR and 97 underwent SG. The analysis of clinicopathological characteristics showed significant differences between the two groups for age, sex, smoking status, %FVC, FEV1.0%, presence of ILD, CTR, and histologic type (Table 1). Age, male sex, smoking, and non-adenocarcinoma were more frequent in the WR group than in the SG group. There were no significant differences in %FEV1.0, presence of chronic obstructive pulmonary disease (COPD), history of CVD, and tumor size between the WR and SG groups. Detailed information regarding the segments resected by SG is presented in Table 2.

**Table 1:** Clinicopathological characteristics according to procedure

	Wedge resection (*n* = 122)	Segmentectomy (*n* = 97)	*p*-value
Age	73 ± 8.0	69 ± 7.4	< 0.01
Age ≥ 80 years	33 (27%)	5 (5.2%)	< 0.01
Sex			
Male (%)	92 (75%)	60 (62%)	0.04
Smoker (current or former)	98 (80%)	62 (64%)	< 0.01
Pulmonary function			
%FVC	104 ± 20	111 ± 19	0.02
%FEV1.0	105 ± 28	110 ± 22	0.12
FEV1.0%	70 ± 12	74 ± 8.9	0.02
Complication			
ILD	16 (13%)	1 (1.0%)	< 0.01
COPD	48 (39%)	28 (29%)	0.12
CVD	15 (12%)	4 (4.1%)	0.05
Tumor			
Size	17 ± 9.2	17 ± 6.1	0.52
CTR			< 0.01
0 to ≤ 0.25	9 (7.4%)	5 (5.2%)	
0.25 to ≤ 0.5	16 (13%)	14 (14%)	
0.5 to < 1.0	20 (16%)	36 (37%)	
CTR = 1.0	77 (63%)	42 (43%)	
Clinical stage			0.04
IA1	48 (39%)	36 (37%)	
IA2	51 (42%)	53 (55%)	
IA3	23 (19%)	8 (8.3%)	
Histologic type			0.01
Ad	89 (73%)	83 (86%)	
Sq	30 (25%)	11 (11%)	
Large cell	0 (0%)	2 (2.1%)	
Adsq	2 (1.6%)	0 (0%)	
Carcinoid	1 (0.8%)	1 (1.0%)	
Pathological factor			
pl(+)	27 (22%)	6 (6.3)	0.01
v(+)	30 (25%)	10 (10)	< 0.01
ly(+)	17 (14%)	4 (4.2)	0.02
Postoperative complications			
Clavien–Dindo ≥ III	3 (2.5%)	6 (6.2%)	0.19
Pulmonary complication	2 (1.6%)	5 (5.2%)	0.25
Postoperative mortality, 30 days	2 (1.6%)	0 (0%)	0.50
Survival			
5-year DFS	67%	88%	< 0.01
5-year LCSS	95%	99%	0.01
5-year OS	81%	91%	< 0.01

Categorical data are described as the number (%). Continuous data are described as the mean** ± **SD

*Ad* adenocarcinoma, *Adsq* adenosquamous cell carcinoma, *CTR* consolidation tumor ratio, *CVD* cardiovascular disease, *DFS* disease-free survival, *ILD* interstitial lung disease, *LCSS* lung cancer-specific survival, *OS* overall survival, *SD* standard deviation, *Sq* squamous cell carcinoma

**Table 2:** Segments resected by segmentectomy

	Part-solid (*n* = 56)	Solid (*n* = 41)
Right		
S^1^	4	0
S^2^	7	2
S^2b+3a^	1	0
S^3^	3	4
S^6^	9	6
S^7+8^	1	0
S^8^	2	3
S^8+9^	1	0
S^9+10^	1	0
S^10^	1	0
Basilary (S^6+7+8+9+10^)	2	3
Left		
S^1+2^	5	2
Upper division (S^1+2, 3^)	8	8
Lingular (S^4+5^)	6	3
S^6^	4	5
S^8^	0	1
S^9^	1	0
Basilary (S^6+8+9+10^)	0	4

Data are described as the number (%)

### Comparison of patient characteristics between tumors with and without GGO

The tumors of 219 patients were classified into two groups: part-solid (tumors with GGO; *n* = 124) and solid (tumors without GGO; *n* = 95). The clinical characteristics of the part-solid and solid tumor groups are presented in Supplementary Table S1. The median CTR was 0.62 in the part-solid group. In comparison with the part-solid group, the solid group was older, showed a male predominance, included a higher proportion of smokers, showed a lower pulmonary function and a higher presence of ILD and COPD, as well as high c-stage, and higher rates of non-adenocarcinoma and pleural and lymphovascular invasion.

### Postoperative survival in all patients

**Fig. 2:**
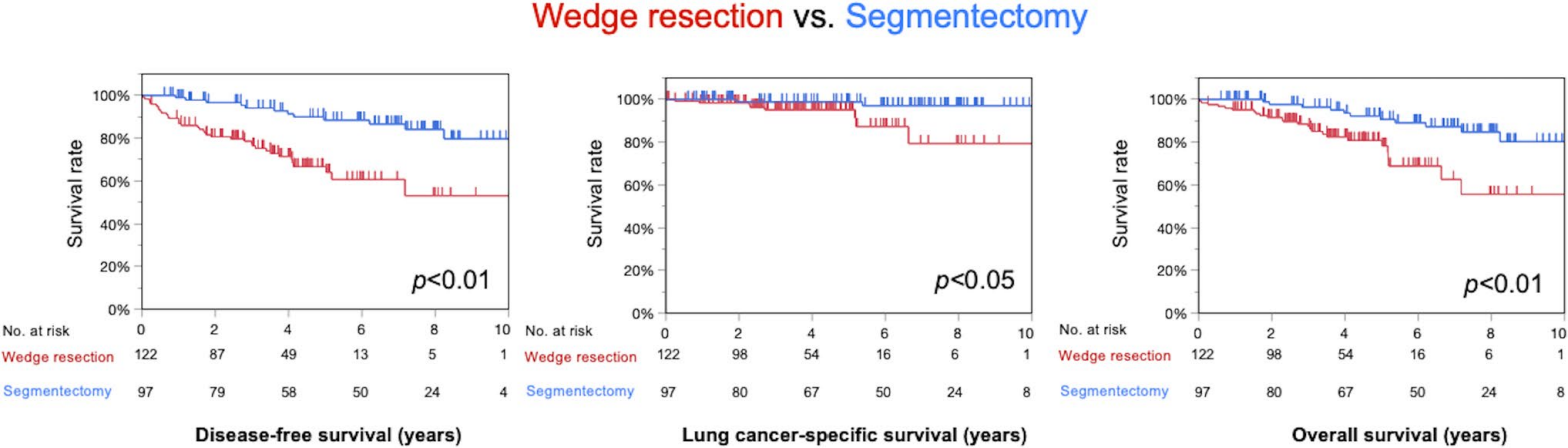
Survival analysis comparing wedge resection (WR) and segmentectomy (SG) in the overall population. WR was significantly worse than SG (WR vs. SG: 5-year disease-free survival, 67% vs. 88% [*p* < 0.01]; 5-year lung cancer-specific survival, 95% vs. 99% [*p* < 0.01]; 5-year overall survival, 81% vs. 91%; *p* < 0.01)

WR was significantly inferior to SG in terms of 5-year DFS (67% vs. 88%; *p* < 0.01), 5-year LCSS (95% vs. 99%; *p* = 0.01), and 5-year OS (81% vs. 91%; *p* < 0.01) (Table 1 and Fig. 2). The median follow-up period was 4.4 years (0.04–13). In contrast, when the part-solid and solid groups were compared, the part-solid group showed significantly superior 5-year DFS (94% vs. 54%; *p* < 0.01), 5-year LCSS (100% vs. 92%; *p* < 0.01), and 5-year OS (95% vs. 71%; *p* < 0.01). (Supplemental Table S1 and Fig. S1).

### Prognostic impact of WR and SG in the part-solid and solid groups

**Fig. 3:**
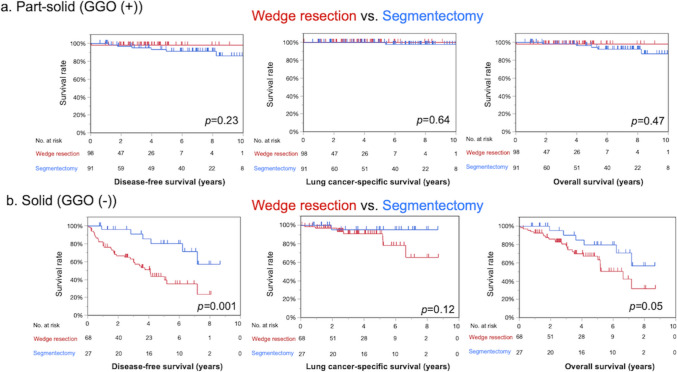
Survival analysis comparing wedge resection (WR) and segmentectomy (SG) in the part-solid and solid subtypes based on CTR. **a** Part-solid group (GGO (+)). In the part-solid group, WR was not inferior to SG in terms of 5-year disease-free survival (DFS), lung cancer-specific survival (LCSS), or overall survival (OS) (DFS, 98% vs. 91% [*p* = 0.23]; LCSS, 100% vs. 100%; OS, 98% vs. 94% [*p* = 0.5]). **b** Solid group (GGO (−)). In the solid group, WR was significantly inferior to SG in terms of DFS and OS (DFS, 43% vs. 80% [*p* < 0.01]; LCSS, 91% vs. 95% [*p* = 0.12]; OS, 67% vs. 80% [*p* = 0.05])

In the part-solid group, WR was equivalent to SG in terms of the 5-year DFS (98% vs. 91%; *p* = 0.23), 5-year LCSS (100% vs. 100%; *p* = 0.64), and 5-year OS (98% vs. 94%; *p* = 0.47) (Fig. 3a). In contrast, in the solid tumor group, WR was significantly inferior to SG in terms of 5-year DFS (43% vs. 80%; *p* < 0.01) (Fig. 3b). Survival in stages IA1-3 is described in Supplemental Figs. S2 and S3. Among patients with solid tumors, recurrence was significantly more frequent in patients who underwent WR than in those who underwent SG (32% vs. 3.7%, *p* < 0.01). In contrast, among patients with part-solid tumors, there was no difference between the WR and SG (0% vs. 4.3%, *p* = 0.26) (Table 3). The recurrence of patients who underwent WR in the solid group was locoregional in 20 of 22 recurrences (91%).

**Table 3:** Recurrence pattern of the first relapse after surgery

	Part-solid (*n* = 124)			Solid (*n* = 95)		
	Wedge resection *n* = 54	Segmentectomy *n* = 70	*p*-value	Wedge resection *n* = 68	Segmentectomy *n* = 27	*p*-value
Recurrence	0 (0%)	3 (4.3%)	0.26	22 (32%)	1 (3.7%)	< 0.01
Locoregional	0 (0%)	3 (4.3%)	0.26	14 (21%)	1 (3.7%)	0.06
Locoregional + distant	0 (0%)	0 (0%)		6 (8.8%)	0 (0%)	0.18
Distant	0 (0%)	0 (0%)		2 (2.9%)	0 (0%)	1.00

Data are described as the number (%)

### Propensity score matching for DFS, LCSS, and OS

**Fig. 4:**
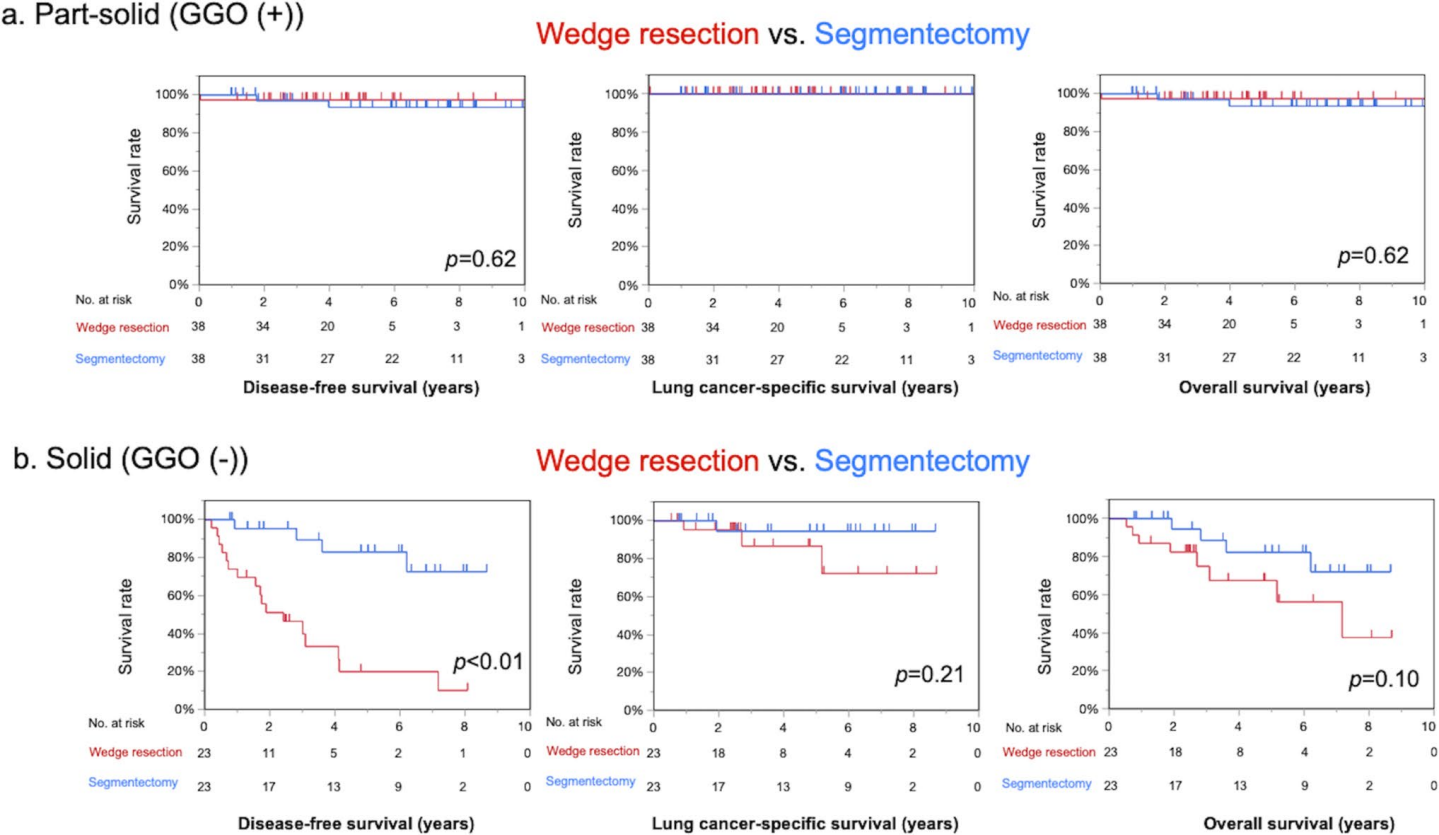
A propensity score-matched analysis for surgical procedure in the part-solid and solid subtypes. **a** Part-solid group (GGO (+)). Wedge resection (WR) was not inferior to segmentectomy (SG) in terms of disease-free survival, lung cancer-specific survival, or overall survival in patients with part-solid tumors. **b** Solid group (GGO (−)). In the solid group, WR was associated with a significantly poorer prognosis in comparison with SG in terms of disease-free survival

A total of patients (76 patients in each group in the part-solid group and 46 in each group in the solid group) were selected after the propensity score analysis (Table 4). In this matched analysis, no recurrence was observed in part-solid GGN, whereas more recurrences were observed in WR than in SG in the solid group. WR was equivalent to SG in terms of DFS, LCSS, and OS in patients with part-solid tumors (*p* = 0.62) (Fig. 4a), whereas WR was significantly inferior to SG in terms of DFS (*p* < 0.01) in patients with solid tumors (Fig. 4b). WR was slightly but not significantly inferior to SG in terms of LCSS and OS.

**Table 4:** Characteristics of 76 patient pairs of the part-solid group and 46 patient pairs of the solid group after propensity score matching for disease-free survival

	Part-solid (*n* = 76)			Solid (*n* = 46)		
	Wedge resection (*n* = 38)	Segmentectomy (*n* = 38)	*p*-value	Wedge resection (*n* = 23)	Segmentectomy (*n* = 23)	*p*-value
Age ≥ 80	3 (7.9%)	2 (5.3%)	1.0	2 (8.7%)	2 (8.7%)	1.0
Male (%)	21 (55%)	20 (53%)	1.0	22 (96%)	20 (87%)	0.61
Smoker	22 (58%)	20 (53%)	0.82	22 (96%)	21 (91%)	1.0
FEV1.0% < 70	8 (21%)	10 (26%)	0.79	14 (61%)	12 (52%)	0.77
ILD	1 (2.6%)	1 (2.6%)	1.0	0 (0%)	0 (0%)	
CVD	3 (7.9%)	3 (7.9%)	1.0	1 (4.3%)	1 (4.3%)	1.0
Tumor						
Size	16 ± 6.2	16 ± 6.2	0.84	16 ± 5.1	15 ± 5.0	0.46
CTR			0.43			
0 to ≤ 0.25	8 (21%)	3 (7.9%)		0 (0%)	0 (0%)	
0.25 to ≤ 0.5	11 (29%)	11 (29)		0 (0%)	0 (0%)	
0.5 to < 1.0	12 (32%)	16 (42%)		0 (0%)	0 (0%)	
CTR = 1.0	7 (18%)	8 (21%)		23 (100%)	23 (100%)	
Pathological factor						
pl(+)	3 (7.9%)	1 (2.7%)	0.61	10 (43%)	4 (17.4%)	0.11
v(+)	1 (2.6%)	0 (0%)	1.00	15 (65%)	8 (35%)	0.08
ly( +)	2 (5.3%)	1 (2.6%)	1.00	7 (30%)	3 (13%)	0.28
Postoperative complications						
Clavien–Dindo ≥ III	1 (2.6%)	3 (7.9%)	0.61	0 (0%)	1 (4.4%)	1.00
Pulmonary complication	0 (0%)	3 (7.9%)	0.24	0 (0%)	1 (4.4%)	1.00
Postoperative mortality, 30 days	1 (2.6%)	0 (0%)	1.00	0 (0%)	0 (0%)	1.00
Clinical stage			1.0			0.80
IA1	26 (68%)	27 (71%)		2 (8.7%)	3 (13%)	
IA2	10 (26%)	10 (26%)		16 (70%)	17 (74%)	
IA3	2 (5.3%)	1 (2.6%)		5 (22%)	8 (17%)	
Recurrence	0 (0%)	0 (0%)		12 (52%)	1 (4.3%)	< 0.01
Locoregional	0 (0%)	0 (0%)		7 (30%)	1 (4.3%)	< 0.05
Locoregional + distant	0 (0%)	0 (0%)		4 (17%)	0 (0%)	0.11
Distant	0 (0%)	0 (0%)		1 (4.3%)	0 (0%)	1.00
Survival						
5-year DFS	97%	94%	0.76	20%	83%	< 0.01
5-year LCSS	100%	100%		87%	94%	0.21
5-year OS	97%	94%	0.62	67%	82%	0.10

Categorical data are described as the number (%)

*CVD* cardiovascular disease, *DFS* disease-free survival, *ILD* interstitial lung disease, *LCSS* lung cancer-specific survival, *OS* overall survival

### Prognostic factors for survival in the study population

A multivariate Cox proportional hazards regression analysis was performed to investigate clinical factors affecting DFS. The univariate analysis revealed that older age (≥ 80 years), male sex, smoking, ILD, solid tumor type, c-stage, and resection type were significantly associated with worse DFS in patients who received WR or SG. In the multivariate analysis, solid type (HR 5.7: 95% confidence interval [CI] 2.3–14; *p* < 0.01) and ILD were identified as independent prognostic factors for worse DFS (Table 5).

**Table 5:** Cox proportional hazards analysis of prognostic factors for disease-free survival

Variables	Univariate analysis		Multivariate analysis	
	HR (95% CI)	*p*-value	HR (95% CI)	*p*-value
Age				
< 80	Reference			
≥ 80	2.9 (1.6–5.4)	0.0007	1.2 (0.6–2.3)	0.63
Sex				
Female	Reference			
Male	4.7 (1.9–12)	0.001	3.4 (0.8–14)	0.09
Smoker				
Never	Reference			
Former or current	3.1 (1.3–7.3)	0.01	0.5 (0.1–2.1)	0.36
FEV1.0%				
≥ 70	Reference			
< 70	1.1 (0.6–2.0)	0.65		
ILD	7.2 (3.4–15)	< 0.001	2.5 (1.1–5.3)	0.02
CVD	0.7 (0.2–2.3)	0.58		
Solid (GGO(−))	12 (5.3–28)	< 0.001	5.7 (2.3–14)	< 0.01
Clinical stage				
IA1	Reference		Reference	
IA2	5.3 (2.1–14)	< 0.001	2.5 (0.9–6.9)	0.08
IA3	10 (3.8–29)	< 0.001	2.7 (0.9–8.6)	0.08
Wedge resection	3.8 (2.0–7.5)	< 0.001	2.1 (0.98–4.5)	0.06

*CVD* cardiovascular disease, *ILD* interstitial lung disease

## Discussion

In the overall population of our study, patients who underwent SG showed significantly better survival than those who underwent WR. However, the effect of WR and SG on survival in patients with part-solid tumors was significantly different from that in patients with solid tumors. Solid-type tumors were independently associated with a worse prognosis.

Among patients with solid-type tumors, recurrence was more frequently found in patients who underwent WR than in those who underwent SG; however, this was not observed in patients with part-solid tumors. Part-solid tumors were associated with significantly better survival and less invasiveness (i.e., low c-stage, dominant adenocarcinoma, and negative pleural and lymphovascular invasion) than solid tumors, which may be related to their superior clinical background (e.g., lower frequency of smoking history). Thus, based on the current data, it can be said that WR was inferior to SG in the overall population and in patients with solid-type tumors, but it was equivalent to SG in patients with part-solid-type tumors with a relatively low malignant status.

In comparison with WR, anatomical SG has the theoretical advantage of achieving appropriate margins and wider resection of the draining lymphatics, including intersegmental planes commonly referenced as a source of residual cancer cells [6, 7]. However, several studies that compared WR and SG have reported different outcomes. Altorki et al*.* described SG and WR as equivalent for peripheral NSCLC < 2 cm in size in a post hoc analysis of CALGB140503 [8] and another retrospective study [9]. In an STS database analysis, WR showed comparable outcomes to SG in patients with c-stage IA disease [10]. In contrast, several meta-analyses have suggested that WR is inferior to SG [11, 12] in stage IA disease. A recent report from the Japanese Joint Committee of Lung Cancer Registry (JJCLCR) database showed that SG was equivalent to WR in patients with cT1a disease, whereas SG was significantly superior to WR in cT1b [13]. A retrospective study by Koike et al*.* also reported that SG significantly improved survival relative to WR in patients with c-stage IA disease [14].

Preoperative HRCT findings of CTR provide great insight into tumor aggressiveness [15, 16]. Hattori et al. reported that adenocarcinoma cases with a CTR of < 1.0 often had a lepidic component and showed a good prognosis and less invasiveness, even if the accompanying GGO was small [17]. This finding is consistent with the results of our study. According to their results, LN metastasis and lymphovascular invasion were rarely found in cases with CTR < 1.0, which suggests that SG, which allows lymphatic dissection [6], might not be necessary for tumors with CTR < 1.0, as long as a sufficient margin can be obtained. However, CTR = 1.0 sometimes causes LN metastasis because patients often have pleural invasion, lymphatic and vessel invasion, and high-grade malignant subtypes [17]. This indicates that WR often causes locoregional recurrence and that SG might be preferable in cases with a CTR of 1.0.

WR is typically performed in high-risk patients. The criteria used to determine whether WR or SG should be performed have not yet been clarified. SG is a superior oncological approach to WR; however, it is more invasive. In the current study, severe postoperative complications (Clavien–Dindo classification ≥ III) were more frequently found in SG than in WR (6.2% vs. 2.4%, *p* = 0.19), especially for pulmonary complications (5.2% vs. 1.6%, *p* = 0.25), despite the worse background of the WR population, although the difference was not significant. In fact, SG was slightly inferior to WR for part-solid tumors in terms of OS (94% vs. 98%), although the difference was not significant. The JCOG 0804 trial showed that WR offers sufficient outcomes for peripheral GGO-dominant tumors if a surgical margin is obtained [3]. Generally, SG requires a larger lung parenchyma resection and longer operation time; therefore, invasiveness and curability should be balanced. A Japanese prospective trial (JCOG1909) to determine the superiority of anatomical segmentectomy over wedge resection in high-risk operable patients with clinical stage IA NSCLC is currently ongoing. Therefore, the results should be carefully reviewed [18].

Our data showed that part-solid (GGO (+)) was an independent good prognostic factor and was well controlled by WR. The CTR is a simple surrogate marker that can be assessed using HRCT alone. Previous Japanese studies classified the CTR in detail (e.g., 0.25 and 0.5) and demonstrated its utility in predicting malignancy [15, 19]; however, it is considered less objective because it is evaluated by only 2-dimensional imaging and there may be overlapping cases (e.g., one evaluator determines that the CTR of a tumor is 0.51, while others determine that it is 0.45). Classification using part-solid (GGO (+)) or solid (GGO (−)) seems to be a simple and reliable method for predicting malignancy and determining the surgical procedure without PET/CT, which is not available in some hospitals. However, the solid type (GGO (−)) contains several pathological subtypes that differ in terms of malignancy. Accordingly, this population requires further studies using other biomarkers or modalities (e.g., PET-CT) to predict tumor invasiveness [20–22].

The present study has several limitations. First, this was a retrospective study, and the selection of patients for either WR or SG was not random. Most sublobar resections in this study were performed because of the high-risk background. The baseline characteristics differed between the WR and SG groups. To reduce this bias, a propensity score-matched analysis was performed after matching for comorbidities. Furthermore, we used LCSS, DFS, and OS, which are closely related to recurrence rates and control for the impact of other causes of death. For the solid type, DFS and recurrence rates were significantly different between WR and SG, whereas differences in LCSS and OS were not statistically significant between WR and SG, even though propensity score-matched analysis was performed. This means that both cancer recurrence and other causes of death were more frequent in the WR group than in the SG group. In fact, it is difficult to verify the intensive indication for limited resection (especially for WR), which was not standard at that time. Therefore, we used real data for the passive indications. Prospective randomized controlled trials are needed to validate these intensive indications. Second, we did not have information on resection margins. Previous studies have shown that a narrow surgical margin (i.e., ≤ 1 cm) is associated with local recurrence [23, 24]. In the current study, locoregional recurrence after SG was favorable (4.1%), which might reflect a relatively wide surgical margin in comparison to WR. Moreover, intraoperative lymph node evaluation by frozen-section examination is rarely performed, especially in cases with a high risk of conversion to lobectomy, because conversion of the surgical procedure to lobectomy is difficult if positive nodes are identified intraoperatively, and shortening of the operation time is considered important. Finally, tumors were not stratified by CTR because the sample size was small, and surgical procedures were determined by patient condition rather than tumor condition in this study. CTR stratification is also important for determining the oncologically optimal surgical procedure (e.g., GGO-dominant T1a tumor should be distinguished from a solid-dominant part-solid T1c tumor). Despite the above limitations, the surgical outcomes after WR and SG in our study were not inferior to those of the previous randomized controlled study [1, 8].

In summary, among patients with c-stage IA peripheral NSCLC who underwent sublobar resection, part-solid cases showed a better prognosis than solid cases. SG was superior to WR for peripheral c-stage IA NSCLC, although WR was equivalent to SG for part-solid-type tumors.

## Supplementary Information

Below is the link to the electronic supplementary material.

Supplementary file1 (DOCX 705 kb)
