# Supplementary material for: Wedge resection versus segmentectomy in peripheral clinical stage IA lung cancer concerning ground-glass opacity
Source: Surg Today. 2025 Sep 22;56(3):301–10. doi: 10.1007/s00595-025-03137-4 (PMC12946241; doi:10.1007/s00595-025-03137-4)
Supplement: Supplementary file 1 — Supplementary file1 (DOCX 705 kb) [file 595_2025_3137_MOESM1_ESM.docx]

**Supplementary Material**

**Supplementary Table**

**Supplementary Table S1.** Clinicopathological characteristics of part-solid (GGO (+)) and solid (GGO (-)) tumors.

**Supplementary Figures**

**Supplementary Figure S1.** Survival analysis of the part-solid and solid groups in the overall population.

**Supplementary Figure S2.** Survival analysis of wedge resection (WR) and segmentectomy (SG) in c-stages IA1–3 in the part-solid group.

**Supplementary Figure S3.** Survival analysis of wedge resection (WR) and segmentectomy (SG) in c-stages IA1–3 in the solid group.

**Supplementary Table S1. Clinicopathological characteristics of the part-solid (GGO (+)) and solid (GGO (-)) tumor groups.**

|  | **Part-solid (n = 124)** | **Solid (n = 95)** | **p-value** |
| --- | --- | --- | --- |
| **Age** | 70 ± 7.8 | 74± 7.8 | <0.01 |
| **Age≥80** | 13(10%) | 25(26%) | <0.01 |
| **Sex** |  |  |  |
| Male (%) | 70 (56%) | 82 (86%) | <0.01 |
| **Smoker** | 75 (60%) | 85 (89%) | <0.01 |
| **Pulmonary function** |  |  |  |
| %FVC | 113 ± 17 | 100± 21 | <0.01 |
| %FEV1.0 | 112 ± 23 | 101 ± 28 | <0.01 |
| FEV1.0% | 74 ± 9.0 | 69 ± 12 | <0.01 |
| **Complication** |  |  |  |
| ILD | 2(1.8%) | 15(16%) | <0.01 |
| COPD | 32(26%) | 44(46%) | <0.01 |
| CVD | 8(6.5%) | 11(12%) | 0.23 |
| **Tumor** |  |  |  |
| Size | 18 ± 9.2 | 16 ± 5.7 | 0.43 |
| CTR |  |  |  |
| CTR 0 to ≤0.25 | 14 (11%) | 0 |  |
| CTR 0.25 to ≤0.5 | 30 (24%) | 0 |  |
| CTR 0.5 to ≤1.0 | 56 (45%) | 0 |  |
| CTR=1.0 | 24 (19%) | 95(100%) |  |
| **Clinical stage** |  |  | <0.01 |
| IA1 | 70 (56%) | 14 (15%) |  |
| IA2 | 45 (36%) | 59 (62%) |  |
| IA3 | 9 (7.3%) | 22 (23%) |  |
| **Operative procedure** |  |  |  |
| Wedge resection | 54 (44%) | 68 (72%) | <0.01 |
| **Histologic type** |  |  | <0.01 |
| Ad | 120 (97%) | 52 (55%) |  |
| Sq | 4 (3.2%) | 37 (39%) |  |
| Large cell | 0 (0%) | 2 (2.1%) |  |
| Adsq | 0 (0%) | 2 (2.1%) |  |
| Carcinoid | 0 (0%) | 2(2.1%) |  |
| **Pathological factor** |  |  |  |
| pl(+) | 4(3.2%) | 29(31%) | <0.01 |
| v(+) | 3(2.4%) | 37(39%) | <0.01 |
| ly(+) | 4(3.2%) | 17(18%) | <0.01 |
| **Survival** |  |  |  |
| 5-year DFS | 94% | 54% | <0.01 |
| 5-year LCSS | 100% | 92% | <0.01 |
| 5-year OS | 95% | 71% | <0.01 |
| Ad, adenocarcinoma; Adsq, Adenosquamous cell carcinoma; CTR, consolidation tumor ratio; CVD, cardiovascular disease; DFS, disease-free survival; ILD, interstitial lung disease; LCSS, lung cancer-specific survival; ly, lymphatic invasion; OS, overall survival; pl, pleural invasion; Sq, squamous cell carcinoma; v, vascular invasion; Categorical data are described as number (%) and continuous data are described as the mean ± SD. | | | |

**Supplementary Figures**


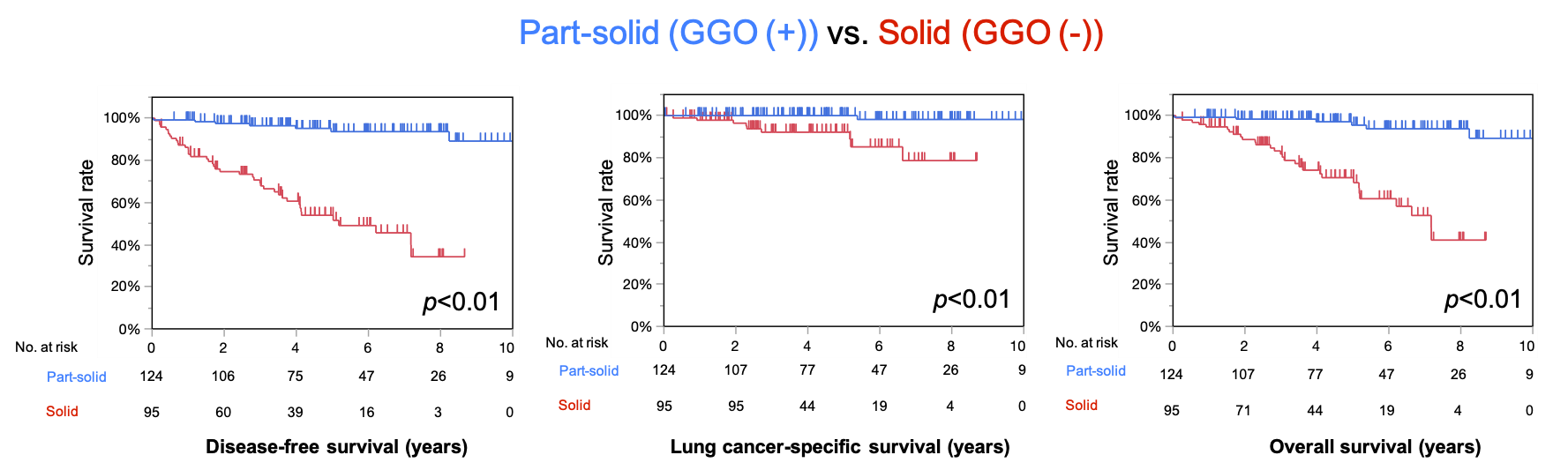


**Supplementary Figure S1.** Survival analysis of the part-solid (GGO (+)) and solid (GGO (-)) tumor groups in the overall population. The solid group showed significantly worse survival than the part-solid group (part-solid vs. solid: 5-year disease-free survival, 94% vs. 54% [*p*<0.01]; 5-year lung cancer-specific survival, 100% vs. 92% [*p*<0.01]; 5-year overall survival, 95% vs. 71% [*p*<0.01]).


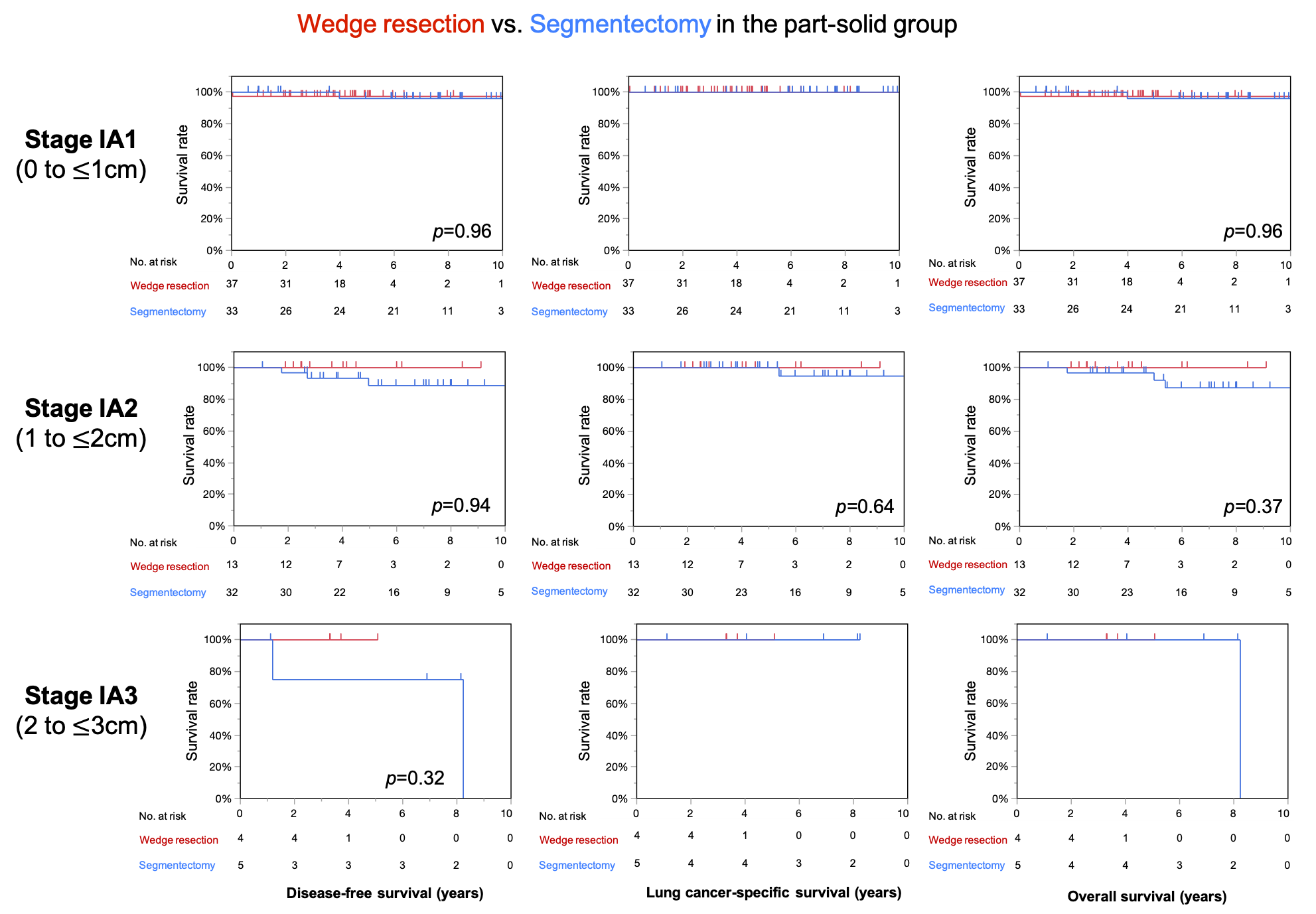


**Supplementary Figure S2.** Survival analysis for wedge resection (WR) and segmentectomy (SG) in c-stages IA1–3 in the part-solid tumor group. There was no significant difference between WR and SG throughout stage IA.


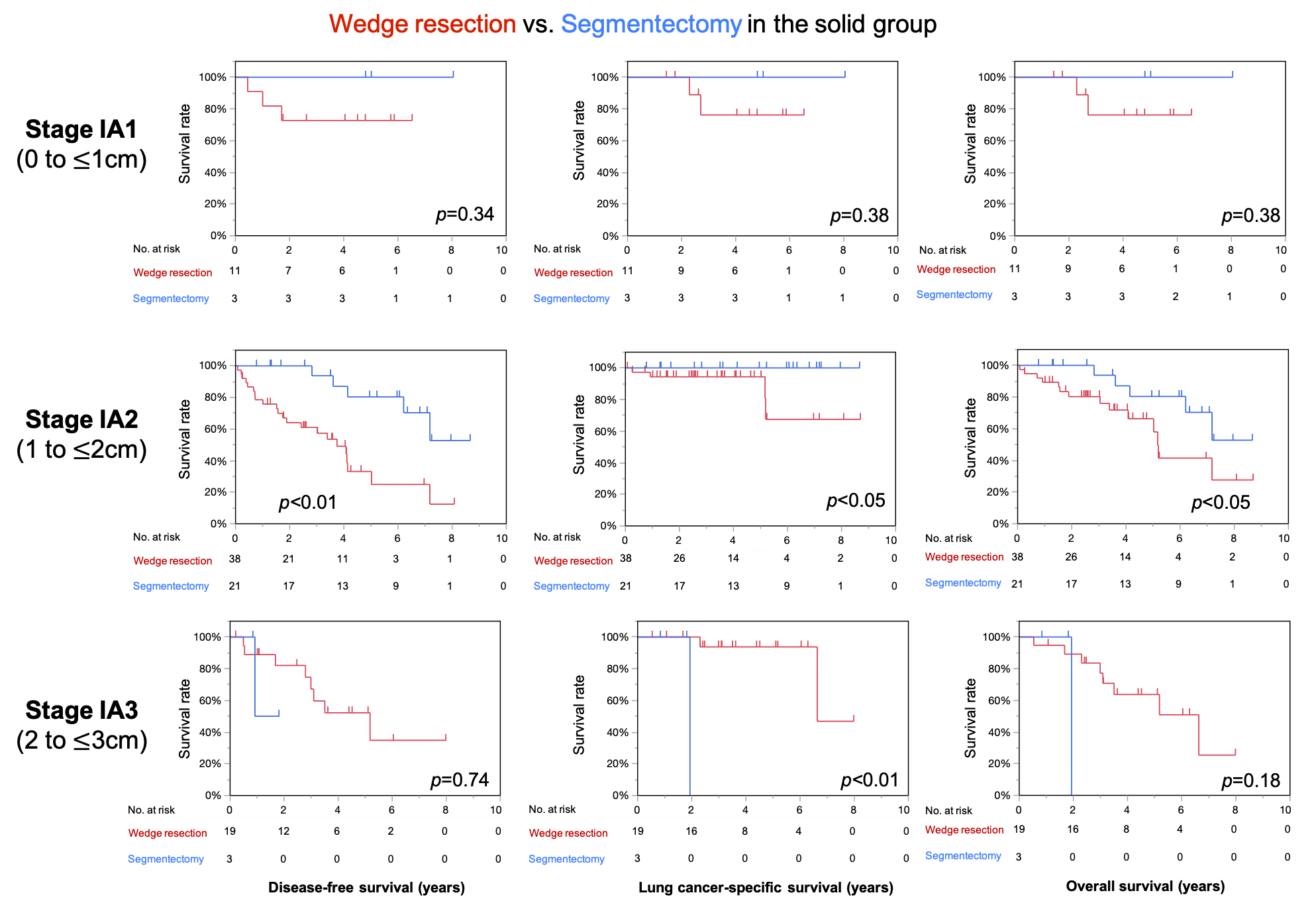


**Supplementary Figure S3.** Survival analysis for wedge resection (WR) and segmentectomy (SG) in c-stages IA1–3 in the solid tumor group.
